# Acknowledgement to Reviewers of *Membranes* in 2017

**DOI:** 10.3390/membranes8010003

**Published:** 2018-01-12

**Authors:** Membranes Editorial Office

**Affiliations:** MDPI AG, St. Alban-Anlage 66, 4052 Basel, Switzerland

Peer review is an essential part in the publication process, ensuring that *Membranes* maintains high quality standards for its published papers. In 2017, a total of 68 papers were published in the journal. Thanks to the cooperation of our reviewers, the median time to first decision was 21.5 days and the median time to publication was 52 days. The editors would like to express their sincere gratitude to the following reviewers for their time and dedication in 2017:
Agrawal, Kumar VaroonKujawa, Joanna AgnieszkaAlbo, JonathanLansman, Jeffry B.Alcoutlabi, MatazLeporatti, StefanoAltmann, FriedrichLi, QianAnderson, David MiltonLi, Robert Kwok-YiuAnsón Casaos, AlejandroLin, HaiqingAntipina, Maria N.Lin, Li-ChiangAramesh, MortezaLlosa Tanco, Margot A.Arnusch, ChristopherLo, Huang-MuArshad, NajlaLu, QuanlongArtyushkova, KaterynaLuo, JiangshuiAvinoam, OriLyonnard, SandrineBabu, RameshMartínez-Monteagudo, Sergio I.Baulin, Vladimir A.Mattheyses, AlexaBayer, Ilker S.Mayer, ChristianBrant, Jonathan A.McGrath, JamesBrinkmann, TorstenMiura, YoshikoBucciantini, MonicaModesti, MicheleBudd, Peter M.Mohankumar, KumaravelCasado-Coterillo, ClaraMotuzas, JuliusChakraborty, SudipNa, HuiminChen, JinhuaNagarajan, SureshbabuCho, Nam-JoonNagy, EndreChun, YoungpilNair, Jijeesh R.Chung, Tai-ShungNakielski, PawelChyau, Charng-CherngNeophytides, StylianosClochard, Marie-ClaudeNestorovich, EkaterinaCooper, GordonNijmeijer, ArianCoronas, JoaquínNonappa, NonappaCui, HaitaoOrchard, SandraDal Santo, VladimiroPályi, GyulaDe Grooth, JorisPazos, CarmenDi Noto, VitoPensabene, VirginiaDixon, Anthony G.Peters, JudithDumee, LudovicPucci, AndreaEaston, Christopher D.Rajca, MariolaEdlund, UlricaRodríguez Ramos, InmaculadaElimelech, MenachemRowley, ChristopherErnst, MathiasRoy, SagarEykens, L.Ruiz-García, AlejandroFan, WeizhengSadrzadeh, MohtadaFatyeyeva, KaterynaSahadevan, RajeshFeng, XundaSchneck, EmanuelFerrari, Maria-ChiaraSelyanchyn, RomanFologea, DanielSentana, IreneGaleazzi, RobertaShah, Jindal K.Garcia Ivars, JorgeSilva, Liana C.Garriga, PereSim, Lee NuangGopalakrishnan, GopanSioutopoulos, DimitriosGorri, DanielSirkar, KamaleshGotthold, DavidSlaughter, GymamaGray, StephenSleutels, TomGu, YibeiSmith, StefanHaddad, MaryamSrinivas, KeerthiHassan, MohammadSzekely, GyorgyHidalgo, ManuelaTachikawa, MasashiHirota, YuichiroTodros, SilviaHong, LiangToy, RandallHou, JingweiTrzcinski, AntoineHu, JackVan Der Bruggen, BartIliev, BoyanVenus, JoachimIto, AkiraWang, TuoJansen, Johannes CarolusWang, XiaohongJi, PengWoldemariam, DanielJin, XinfangWong, Him ChengJohn, Vijay T.Wu, BingKaneko, TakashiXiang, YuanKang, Sang WookXin, RanKarakaya, CananYabu, HiroshiKentish, SandraYoung, StephanieKertèsz, SzabolcsYu, LeonKiebach, Wolff-RagnarZha, ShangwenKim, JeonghwanZhang, PengKobayashi, ShunsukeZhu, ShanKoibuchi, Hiroshi


